# ABO blood type correlates with survival on prostate cancer vaccine therapy

**DOI:** 10.18632/oncotarget.4993

**Published:** 2015-08-18

**Authors:** Saddam M. Muthana, James L. Gulley, James W. Hodge, Jeffrey Schlom, Jeffrey C. Gildersleeve

**Affiliations:** ^1^ Chemical Biology Laboratory, Center for Cancer Research, National Cancer Institute, National Institutes of Health, Frederick, MD, USA; ^2^ Chemistry Department, College of Science & General Studies, Alfaisal University, Riyadh, KSA; ^3^ Genitourinary Malignancies Branch, Center for Cancer Research, National Cancer Institute, National Institutes of Health, Bethesda, MD, USA; ^4^ Laboratory of Tumor Immunology and Biology, Center for Cancer Research, National Cancer Institute, National Institutes of Health, Bethesda, MD, USA

**Keywords:** cancer vaccine, immunotherapy, PROSTVAC-FV, Glycan microarray, blood type

## Abstract

Immunotherapies for cancer are transforming patient care, but clinical responses vary considerably from patient to patient. Simple, inexpensive strategies to target treatment to likely responders could substantially improve efficacy while simultaneously reducing health care costs, but identification of reliable biomarkers has proven challenging. Previously, we found that pre-treatment serum IgM to blood group A (BG-A) correlated with survival for patients treated with PROSTVAC-VF, a therapeutic cancer vaccine in phase III clinical trials for the treatment of prostate cancer. These results suggested that ABO blood type might influence efficacy. Unfortunately, blood types were not available in the clinical records for all but 8 patients and insufficient amounts of sera were left for standard blood typing methods. To test the hypothesis, therefore, we developed a new glycan microarray-based method for determining ABO blood type. The method requires only 4 μL of serum, provides 97% accuracy, and allows simultaneous profiling of many other serum anti-glycan antibodies. After validation with 220 healthy subjects of known blood type, the method was then applied to 74 PROSTVAC-VF patients and 37 control patients from a phase II trial. In this retrospective study, we found that type B and O PROSTVAC-VF patients demonstrated markedly improved clinical outcomes relative to A and AB patients, including longer median survival, longer median survival relative to Halabi predicted survival, and improved overall survival via Kaplan-Meier survival analysis (*p* = 0.006). Consequently, blood type may provide an inexpensive screen to pre-select patients likely to benefit from PROSTVAC-VF therapy.

## INTRODUCTION

Immunotherapies, treatments that use a person's own immune system to fight disease, are transforming cancer care. Immunotherapies can provide long-lasting, durable responses, and several have received U.S. Food and Drug Administration (FDA) approval recently. While efficacious, clinical responses vary considerably from patient to patient. Some patients live years longer than expected while other seemingly similar patients have little or no apparent benefit. Strategies to target immunotherapies to likely responders would allow patients to make better treatment decisions, leading to improved outcomes and reduced health care costs [1–4]. Unfortunately, identification of reliable predictive biomarkers has proven challenging.

One promising immunotherapy is PROSTVAC-VF, cancer vaccine in phase III clinical trials for the treatment of advanced prostate cancer.[5] PROSTVAC-VF induces immunity to prostate-specific antigen (PSA) using genetically modified vaccinia and fowlpox encoding PSA and three costimulatory molecules (LFA-3, B7.1, and ICAM-1) [6]. Although PROSTVAC-VF treatment was associated with preliminary evidence of an 8 to 9 month improvement in median overall survival in two phase II clinical trials [7, 8], not all patients experience improved survival. In a recent study, we reported that pre-vaccination IgM antibody levels to blood group A trisaccharide (BG-A_tri_) significantly correlate with survival [9]. These antibodies bind to the BG-A antigen displayed on the surface of the PROSTVAC-VF poxvirus vectors. The importance of immune recognition of an ABO antigen raised the possibility that a patient's blood type may influence their response to the vaccine; however, correlations between serum IgM to BG-A and survival are insufficient to establish a correlation between blood type and survival. More specifically, correlations between blood group antibodies and blood type tend to be weaker for IgM than IgG antibodies and tend to be weaker for trisaccharides in comparison with larger glycan structures. In fact, some patients with type A or AB blood have relatively high levels of IgM to BG-A_tri_. Thus, we needed to evaluate potential correlations between survival and ABO blood type directly. Unfortunately, blood type information was not available in the clinical records for most of these patients. Therefore, we needed to obtain blood type information experimentally. Standard methods based on agglutination of red blood cells require large amounts of sera, and DNA analysis requires whole blood [10, 11]. Unfortunately, we only had very small amounts of sera (5–20 μL) for the PROSTVAC-VF patients from this study and additional blood draws were impossible as most patients are now deceased. Therefore, an alternative method was needed. It should be noted that many clinical studies and epidemiological studies store only small amounts of sera/plasma for retrospective analysis. Therefore, new methods for blood typing that use miniscule amounts of serum/plasma could be useful in a variety of studies.

**Figure 1:**
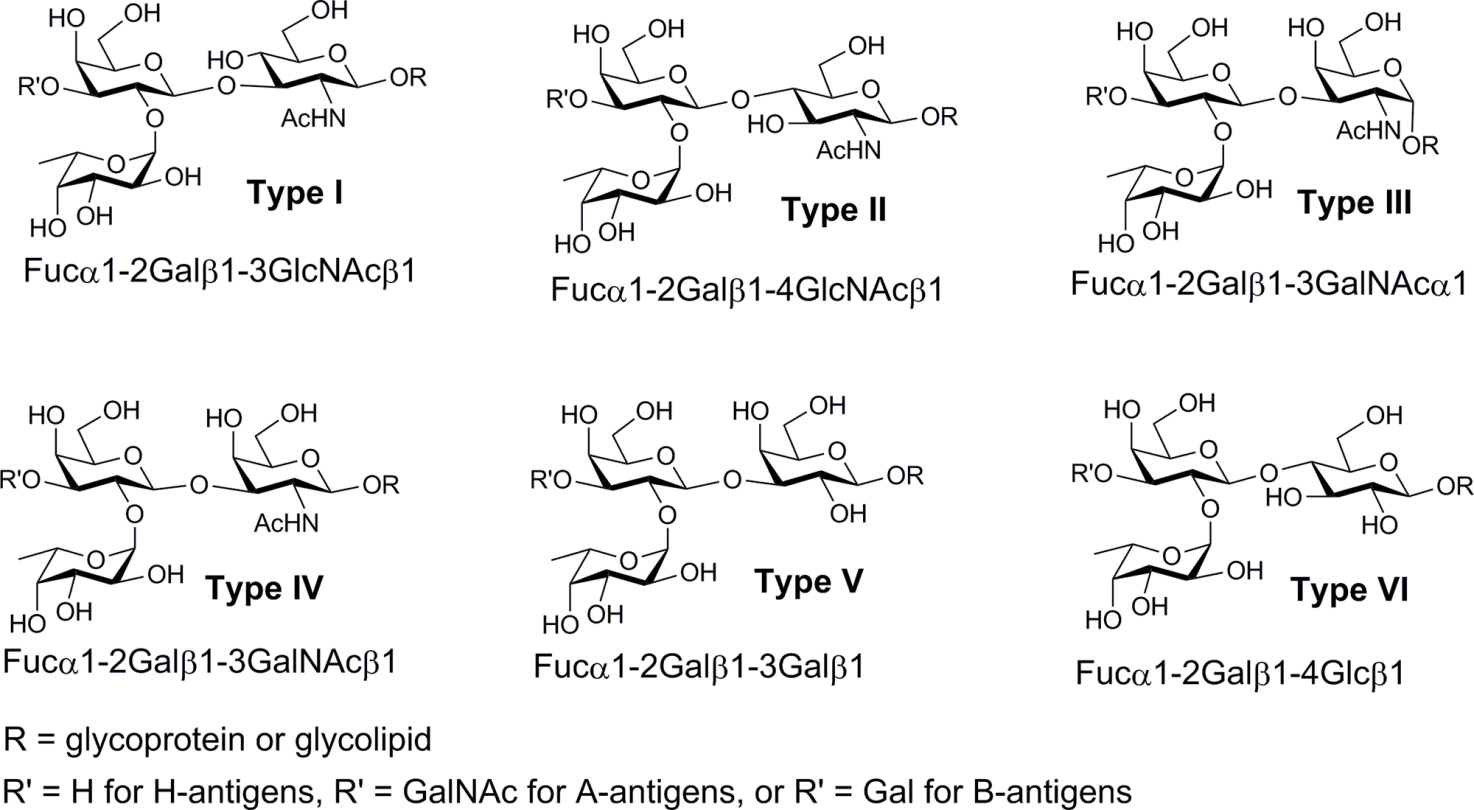
Structures of the different types of blood group A and B tetrasaccharide determinants (type 1 through type 6) and the six blood group H trisaccharides

In principle, blood type can be determined by evaluating the presence or absence of serum antibodies to blood group antigens (i.e., reverse typing). The antigens that define ABO blood types are glycans that are displayed on the surfaces of erythrocytes and other cells [12, 13]. Blood group A (BG-A) and B (BG-B) antigens that are not expressed on an individual's own cells are viewed as foreign by their immune system, resulting in the production of antibodies to the corresponding glycans. While reverse typing is simple conceptually, identifying optimal glycans for antibody detection is not trivial [14]. The BG-A and BG-B determinants are defined as trisaccharides [BG-A has the terminal sequence GalNAca1-3(Fuca1-2)Gal; BG-B has the terminal sequence Gala1-3(Fuca1-2)Gal]. As will be illustrated below, the simple trisaccharides are not suitable capture agents for blood typing with high accuracy. In nature, however, these trisaccharides are appended to various carrier glycan chains resulting in 12 different tetrasaccharides (BG-A1 through A6 and BG-B1 through B6, see Figure 1). The BG-A and BG-B determinants are biosynthesized from 6 different blood group H trisaccharides (BG-H1 through H6, see Figure 1). Previous studies have detected chain type specific antibodies to BG-A and BG-B glycans in human serum [15, 16], indicating that chain structure can influence the ability to capture some antibodies to blood group antigens. Moreover, the ABH determinants can be presented in different contexts on the cell surface (e.g., on lipids or proteins; at high or low density). It was not clear which structure(s) and presentation(s) would be best for detecting the relevant antibody populations.

Glycan microarray technology is a powerful high-throughput tool for studying interactions of hundreds of carbohydrates with a variety of macromolecules [17–20]. Glycan array assays require only tiny amounts of sera/plasma (2–5 μL) and provide the opportunity to examine many combinations of glycan structure and presentation in a single experiment. In this study, we used a glycan array containing 380 array components, of which 71 are variations of ABH determinants. IgG and IgM antibodies to BG-A and BG-B determinants were evaluated and used to develop a microarray-based method for blood typing. Next, we applied this method to obtain blood types for patients in the PROSTVAC-VF study and found that patient's blood type does correlate with survival. Given that blood type can be easily obtained for future studies using standard methods, blood type may provide a simple and cost effective approach for selecting patients likely to benefit from PROSTVAC-VF therapy.

## RESULTS

### Development of an array-based blood typing method and experimental design

To the best of our knowledge, blood typing has not been reported previously using a glycan microarray. In the first phase of this project, we developed a glycan microarray-based assay for assigning blood type using serum samples from healthy individuals of known blood type. Reverse blood typing involves evaluating the presence or absence of serum antibodies to ABO antigens. In principle, individuals lacking a particular ABO antigen on their red blood cells will have antibodies directed against it in their sera/plasma, and individuals expressing a particular ABO antigen will have no antibody directed against it. In practice, however, selectively capturing the correct serum antibodies with synthetic glycans is not trivial. Therefore, an optimal method needed to be determined experimentally. In particular, we needed to optimize (a) the number of array components, (b) the structures and presentations of those glycans, and (c) the use of IgG and/or IgM signals. The microarray based strategy allows one to investigate many possible variations of structure and presentation as well as many different combinations of 2 or more determinants. For each method, there were two key criteria: the proportion of subjects that can be classified and the accuracy of those classifications. Our goal was to optimize both factors with an emphasis on accuracy over classification rate. To ensure consistency, sera were divided into three sets (see Supporting Information, Table S1): a training set (*n* = 60), a test set (*n* = 40), and a validation set (*n* = 120). The initial methods were developed using the training set and then refined with the test set. The best method was then further evaluated with the validation set.

### Antibody profiles from the training set

The training set (*n* = 60) was used to identify array components with high correlations to blood type in healthy subjects. Heat maps provide an overall view of IgG and IgM anti-glycan antibody signals to blood group A and B antigens (Supporting Information, Figure S1). In general, IgG signals were more strongly correlated with blood type than IgM. Correlations for tetrasaccharides tended to be better than trisaccharides (see Table 1 and Figure S2). The strongest correlations for IgG were observed for blood group antigens with type 1–4 chains (Table 1). For IgM antibodies, the strongest correlations were for type 2 A and B tetrasaccharides. Overall, stronger correlations between IgG signals and blood type were observed with higher density glycans, while some lower density glycans showed better correlations for IgM. Antibody signals were not affected by up to three freeze-thaw cycles.

**Table 1:** Correlations between antibody signals and blood type in healthy subjects

	Training Set (*n* = 60)		Training Set (*n* = 60)
Capture Antigen	IgG (*p* values)	Capture Antigen	IgM (*p* values)
BG-A3-Oct-14	4.77E-28	A-LeB hexa - 06	1.94E-17
BG-A1-Oct-12	7.01E-21	2′F-A type 2-Sp - 05	9.77E-15
BG-A4-Oct-14	7.18E-21	2′F-A type 2-Sp - 13	9.91E-15
BG-A4-Oct-05	3.74E-20	BG-A2-Oct-04	5.23E-14
Globo A - 09	4.54E-20	BG-B2-Sp - 05	1.71E-13
BG-A3- Oct-04	9.83E-19	BG-A2-Sp - 05	3.76E-13
2′F-A type 2-Sp - 13	2.28E-17	BG-A2-Sp - 07	9.95E-13
BG-A2-Oct-16	3.00E-17	BG-A3-Oct-04	1.92E-12
BG-A1-Sp - 15	4.11E-16	BG-A3-Oct-14	6.28E-12
BG-A1-Sp - 05	5.74E-16	BG-A2-Sp - 17	1.05E-11
Globo A - 03	2.94E-15	BB-B2-Sp - 07	2.82E-11
BG-A5-Oct-05	6.90E-15	BG-A2-Oct-16	3.48E-11
BG-A1_penta_ -05	7.57E-15	BG-B2-Oct-17	6.67E-10
BG-A2-Sp - 17	1.44E-14	BG-A4-Oct-14	1.32E-09
2′F-A type 2-Sp - 05	3.55E-14	BG-A4-Oct-05	2.20E-09
BG-A2-Sp - 07	1.32E-13	BG-B2-Sp - 20	2.89E-09
BG-B2-Oct-17	1.39E-13	BG-B6-Oct-15	3.19E-09
BG-A2-Sp - 05	1.64E-13	BG-B2-Oct-03	4.87E-09
BG-A5-Oct-16	1.68E-13	BG-A1-Sp - 15	4.13E-08
BG-A1-Oct-04	2.01E-13	BG-A1-Sp - 05	4.39E-08
A-LeB hexa - 06	2.19E-13	BG-A6-Oct-23	6.12E-08
BG-B3-Oct-17	6.74E-13	BG-B1-Sp - 16	1.32E-07
BG-B1-Sp - 16	2.72E-12	BG-A1-Oct-12	1.40E-07
BG-B1-Oct-15	4.48E-12	BG-A5-Oct-05	2.51E-07
BG-A2-Oct-04	7.08E-12	BG-B1-Sp - 04	3.41E-07
Globo B - 12	1.09E-11	BG-A5-Oct-16	4.08E-07
BG-B4-Oct-06	2.02E-11	2′F-B type 2-Sp - 07	5.06E-07
BG-B3-Oct-05	3.84E-11	BG-A1_penta_ -05	1.27E-06
BG-B_tri_ - 13	4.27E-11	BG-B6-Oct-03	3.14E-06
BG-B5-Oct-17	5.25E-11	BG-B1-Oct-15	4.53E-06
BG-B2-Sp - 05	8.75E-11	BG-A1-Oct-04	1.44E-05
Globo B - 05	8.81E-11	BG-A6-Oct-04	7.51E-05
BG-B4-Oct-16	1.87E-10	BG-A_tr_i -19	1.17E-04
BG-B1-Sp - 04	4.28E-10	BG-B_tri_ - 13	1.38E-04
BG-B2-Sp - 20	5.62E-10	BG-B1-Oct-05	3.34E-04
BG-A6-Oct-23	6.35E-10	2′F-B type 2-Sp - 03	3.54E-04
BG-B2-Oct-03	6.66E-10	Globo A - 03	5.69E-04
BG-B2-Sp - 07	7.33E-10	2′F-B type 2-Sp - 15	5.79E-04
BG-A_tri_ -19	9.51E-10	BG-B3-Oct-05	1.52E-03
BG-B5-Oct-04	1.47E-09	Globo B - 12	2.53E-03
2′F-B type 2-Sp - 07	2.53E-08	BG-B5-Oct-04	3.54E-03
2′F-B type 2-Sp - 15	3.05E-08	BG-B4-Oct-06	5.12E-03
BG-A6-Oct-04	3.02E-07	Globo B - 05	6.87E-03
BG-B1-Oct-05	5.75E-07	Globo A - 09	7.20E-03
2′F-B type 2-Sp - 03	1.42E-06	BG-B4-Oct-16	1.13E-02
BG-B6-Oct-15	4.22E-06	BG-B5-Oct-17	2.79E-02
BG-B6-Oct-03	3.72E-03	BG-B3-Oct-17	3.17E-02

^a^For a full description of array components, see the full microarray data deposited in National Center for Biotechnology Information's (NCBI) Gene Expression Omnibus, accession number GSE68405. “Sp” and “Oct” are different linkers used to attach the glycans to bovine serum albumin. The number after the abbreviation refers to the average number of epitopes per molecule of albumin (averages <8 are considered low density and averages >8 are considered high density).

Based on the profiles, 4 of the 60 samples appeared to have vastly different antibody profiles than expected based on the listed blood type. These samples, along with 21 other samples, were blinded and then re-typed using standard methods. Based on the results, all four samples appear to be mislabeled (see Supporting Information, Table S2 for details). For the purposes of this study, we report the accuracies of our method for both the original assignments provided by the vendor and the new assignments based on re-typing.

### Initial development and evaluation of blood typing methods using the training set

#### Two component systems

The simplest methods for blood typing used two array components (an A antigen and a B antigen) to assign blood type. Based on the correlations described above, about 140 different combinations of two components were evaluated. For each component, the signals were divided into three ranges: positive, negative, and unclassified. The thresholds were varied to optimize accuracy while still classifying at least 80% of the samples. For all methods tested, the blood typing accuracies using a two-component system ranged from about 60% to 90%. As anticipated, only modest accuracies (57–77%) could be achieved using the BG-A and BG-B trisaccharides (see Table 2). The best classification rate and accuracy for a two-component system was achieved using IgG antibody signals to the type 3 tetrasaccharides, BG-A3-Oct-14 and BG-B3-Oct-17 (classification rate = 90% and accuracy = 89–93%). In general, IgM signals provided lower classification rates and accuracies than IgG. For example, the accuracy using IgM signals to BG-A and BG-B trisaccharides was about 57–60%, as compared to 75–77% for IgG. The best classification rate and accuracy for IgM were obtained using type 2 tetrasaccharides (BG-A2-Sp-17 and BG-B2-Sp-05), with a classification rate of 97% and an accuracy of 83–88%. Glycan density had a significant impact on correlations with blood type and success in blood typing. In general, better sensitivity and specificity was obtained using IgG signals to higher density glycans, while lower density glycans tended to be better using IgM signals.

**Table 2:** Selected blood typing results using two-, four-, and 10-component systems

Training Set (*n* = 60) BG-A Antigens	BG-B Antigens	Number of Classified	Number of Unclassified	Accuracy (Uncorrected)^1^	Accuracy (Corrected)^1^
**Two components**					
BG-A_tri_- 19 (IgG)	BG-B_tri_ – 13 (IgG)	52 (87%)	8 (13%)	39/52 (75%)	40/52 (77%)
BG-A_tri_- 19 (IgM)	BG-B_tri_ – 13 (IgM)	60 (100%)	0 (0%)	32/60 (57%)	36/60 (60%)
BG-A3-Oct-14 (IgG)	BG-B3-Oct-17 (IgG)	54 (90%)	6 (10%)	48/54 (89%)	50/54 (93%)
BG-A2-Sp-17 (IgG)	BG-B2-Sp-05 (IgG)	60(100%)	0 (0%)	52/60 (87%)	53/60 (88%)
BG-A2-Sp-17 (IgM)	BG-B2-Sp-05 (IgM)	58 (97%)	2 (3%)	48/58 (83%)	52/58 (90%)
**Four components**					
BG-A2-Sp -17 (IgG) BG-A2-Sp -17 (IgM)	BG-B2-Sp-20 (IgG) BG-B2-Sp-05 (IgM)	44 (73%)	16 (27%)	43/44 (98%)	44/44 (100%)
BG-A3-Oct-14 (IgG) BG-A2-Sp -17 (IgM)	BG-B3-Oct-17 (IgG) BG-B2-Sp -05 (IgM)	51 (85%)	9 (15%)	48/51 (94%)	51/51 (100%)
**10 Components**					
Flow Chart (Figure 2)	Flow Chart (Figure 2)	57(95%)	3 (5%)	53/57 (93%)	57/57 (100%)

1 Uncorrected refers to the accuracy based on the vendor's original assignment of blood type; corrected refers to the accuracy based on our re-assignments of 4 samples (see Methods section and Supporting Information, Table S2).

#### Three or more component systems

To further improve the accuracy of our typing methods, we next evaluated about 50 combinations of three and four components system using both IgG and IgM antibody levels. In general, the four-component systems were able to obtain blood type with accuracies greater than 95%, but many combinations resulted in a higher number of unclassified subjects given that both BG-A components and both BG-B components must be in agreement to assign a blood type to a sample. A four component system using the IgG levels to BG-A3-Oct-14 and BG-B3-Oct-17 and the IgM levels to BG-A2-Sp-17 and BG-B2-Sp-05 was one of the best combinations. Using this set of glycans, we were able to classify 85% of subjects with 94–100% accuracy (Table 2).

**Figure 2:**
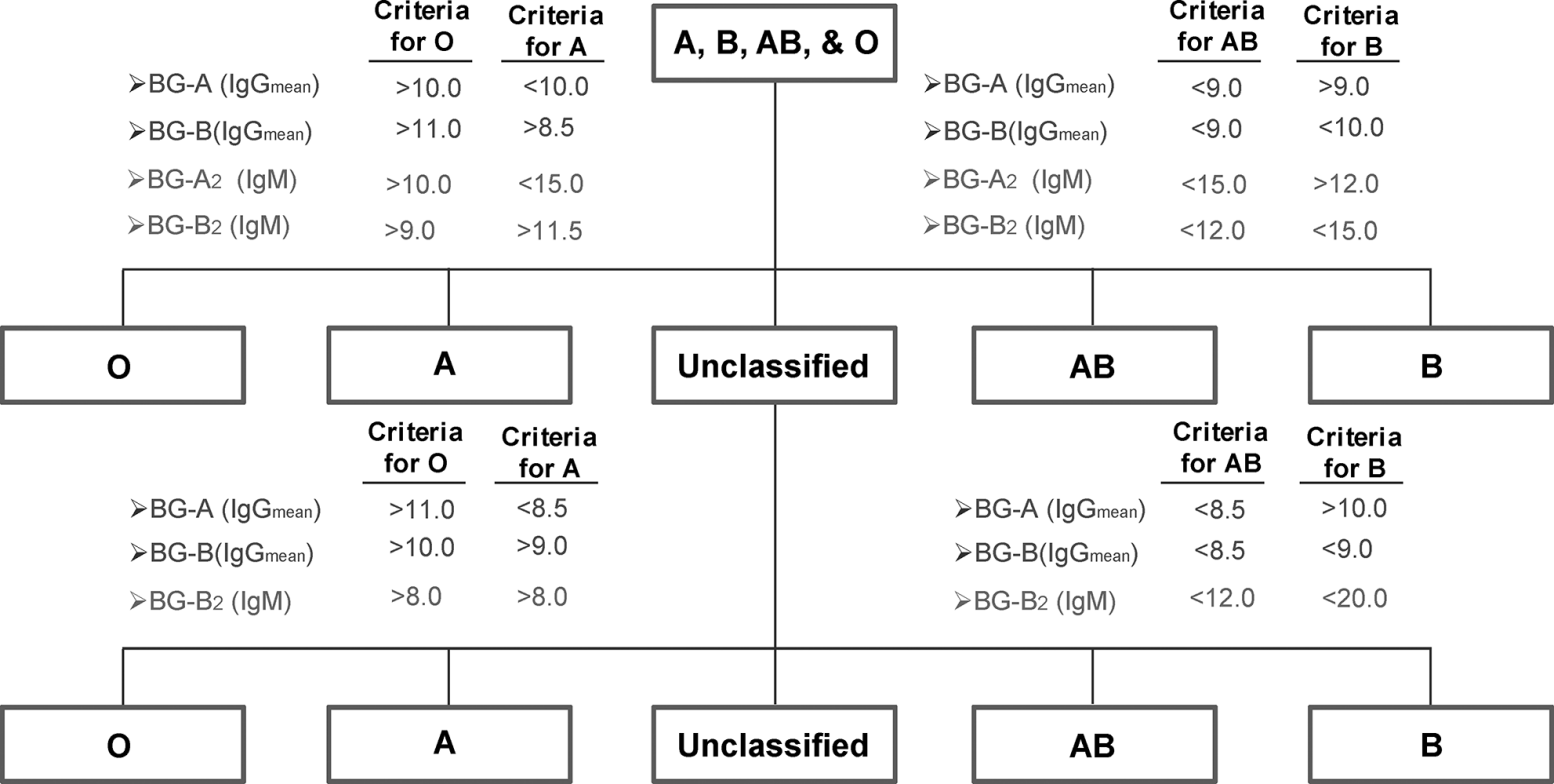
Flow chart for blood typing using the 10-component, 2-step method The first stage of blood typing involves determining the average IgG signals to four BG-A components (BG-A2-Sp-17, BG-A2-Oct-17, BG-A3-Oct-14, and Globo A -09), the average signal to four BG-B components (BG-B2-Sp-20, BG-B2-Oct-17, BG-A3-Oct-17, and Globo B -12), and IgM signals to BG-A2-Sp-17 and BG-B2-Sp-05 2–05. For classification, the 4 threshold values listed for each blood type must be satisfied. Samples that are unclassified after the first stage are then re-evaluated in a second stage using the listed criteria.

After evaluating various methods and different component systems, we found that blood type can be consistently obtained with highest accuracy using a 10-component system. Blood group A antibodies were assessed by evaluating the IgM signals to BG-A2-Sp-17 and the average IgG signal to four glycans (BG-A2-Sp-17, BG-A2-Oct-16, BG-A3-Oct-14, and Globo A-09). Blood group B antibodies were assessed by evaluating the IgM signals to BG-B2-Sp-05 and the average IgG signal to four glycans (BG-B2-Sp-20, BG-B2-Oct-17, BG-B3-Oct-17, and Globo B-12). Using this method, we were able to classify 55 out of 60 subjects (92%) while five subjects (8%) were not classified. The blood types for all of the 55 subjects classified were assigned with 93% or 100% accuracy based on vendor original assignment or after retyping suspected samples, respectively. To further improve the classification rate, we implemented a second step (see flow chart, Figure 2) to assign blood types of the unclassified subjects that uses more stringent thresholds and fewer glycans. Using the two-step process to assign blood types for the training set (*n* = 60), we were able to classify 57 (95%) out of the 60 subjects with 93–100% accuracy.

### Additional evaluation and validation

Next, our best methods (three two-component systems, two four-component systems, and one 10-component system) were evaluated in a test set (*n* = 40). The rate of classification and accuracy for the tested methods were similar to that of the training set (Supporting Information, Table S3). The three two-component systems produced accuracies of 85–91%. The two four-component systems produced higher accuracies (100%), but the rates of unclassified samples were also high (17–20%). Using our best method (10 component system with second step for unclassified samples), we were able to assign blood type to 39 out of 40 subjects with 100% accuracy.

Given the success of the 10-component method in the training and test sets, this system was selected for additional evaluation in a larger blinded validation set (*n* = 120). Samples from two different vendor sources were included in the validation study. Out of the 115 subjects classified, 112 were accurately classified (97.4%) while three subjects were inaccurately classified (2.6%). Thus, the 10-component system provided consistently high accuracies and classification rates in each of the sample sets. Also, there were no statistically significant differences in accuracy or classification rate between the two vendors. Therefore, this method was selected for analysis of the samples from the PROSTVAC-VF patients.

### Application of the blood typing method to PROSTVAC-VF patients

The PROSTVAC-VF samples were profiled using a smaller, focused array containing all the BG-A and BG-B determinants. PROSTVAC samples (*n* = 74) and control subjects vaccinated with empty vector (*n* = 37) were run in randomized order to minimize artifacts due to day-to-day variation. Using our method, we were able to assign blood types to 72 out of 74 available PROSTVAC-VF samples and all 37 controls (see Supporting Information, Tables S4 and S5). In addition to these assignments, blood types for eight additional PROSTVAC-VF patients were available from clinical records for a total of 80 PROSTVAC-VF patients and 37 controls. The proportions of each blood type from our group were similar to the expected proportions for the United States population (see Supporting Information, Table S4), implying that there were no major problems with the samples (e.g. degradation).

### Correlations between blood type and survival on PROSTVAC-VF

**Figure 3:**
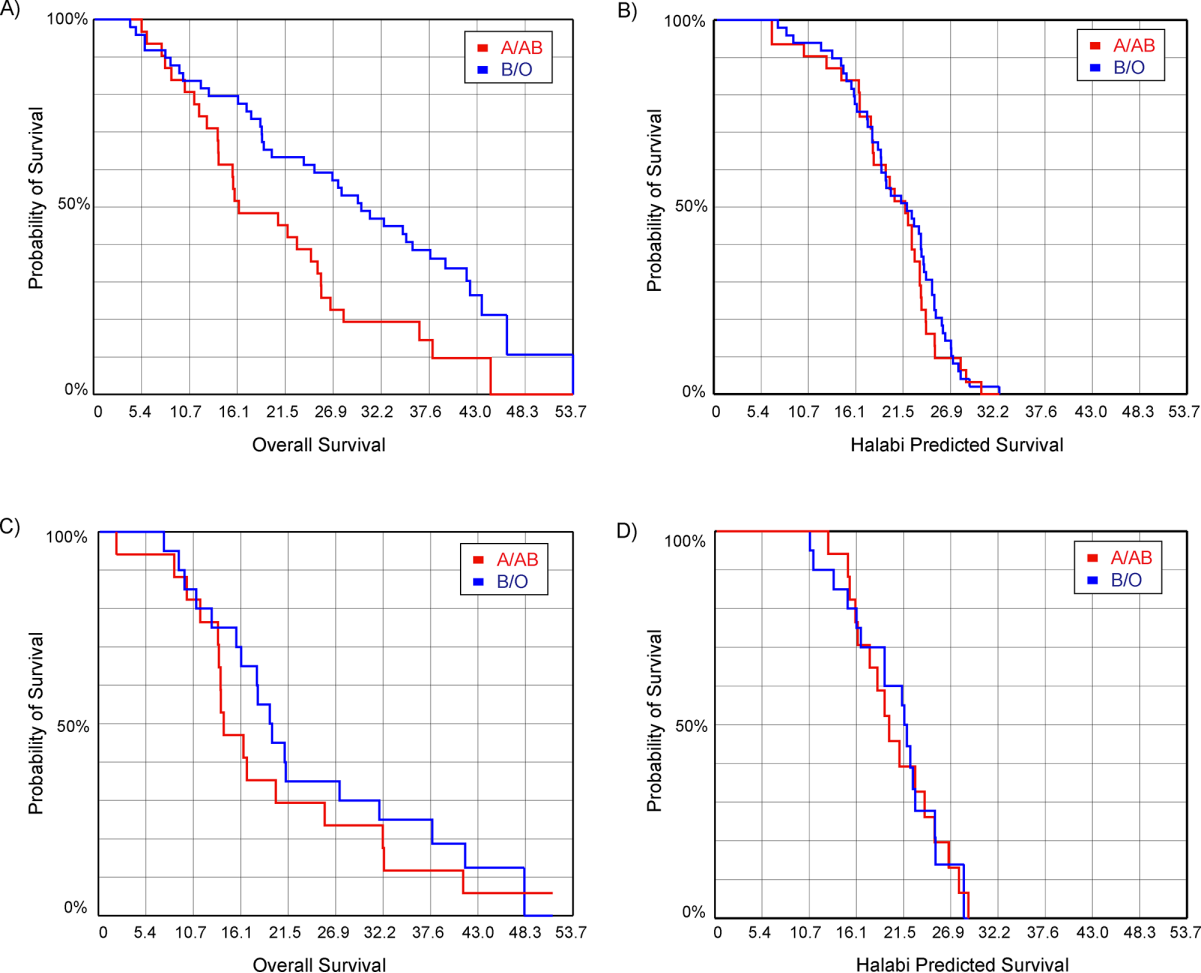
Kaplan-Meier survival curves evaluating the influence of blood type **A.** overall survival for prostate cancer patients treated with PROSTVAC (*p* = 0.006); **B.** Halabi predicted survival for patients treated with PROSTVAC (*p* = 0.53); **C.** overall survival for patients in the control arm (*p* = 0.41); **D.** Halabi predicted survival for patients in the control arm (*p* = 0.98).

Next, we retrospectively evaluated potential survival correlations with blood type in both PROSTVAC-VF patients and controls. It should be noted that the proportions of each blood type for the PROSTVAC-VF patients and controls were similar (see Supporting Information, Table S4), indicating that there were no major imbalances between these groups in terms of blood type. For the PROSTVAC-VF patients, significant differences in survival were observed based on blood type. Blood type B patients had the longest median survival (median = 38 months, *n* = 8), followed by type O (median = 27.6 months, *n* = 41), type AB (median = 21.7 months; *n* = 7), and type A (median = 15.8 months, *n* = 24). For comparison, the median survival for all 80 PROSTVAC-VF patients in this study was 24.9 months; median survival for the 37 control patients was 17.9 months. Given the small numbers of AB and B patients, statistical analyses with these groups were not practical. Therefore, we combined type O and B patients together and type A and AB patients together to form two groups: patients in which the blood group A antigen is self (A/AB) and patients in which blood group A is foreign/non-self (B/O). The median survival for B/O patients was 30 months, compared to 16.3 months for A/AB patients; the difference was statistically significant (*p* = 0.03). These groups were also compared using the Kaplan-Meier survival analysis (see Figure 3A). Type B/O patients had a statistically significant improvement in survival (*p* = 0.006). Finally, we calculated the odds ratio (OR) that a PROSTVAC-VF patient would live longer than the median survival for the full group (24.9 months). Type B/O patients were about 2.5 times more likely than A/AB patients to live longer than 24.9 months (OR = 2.54; 95% confidence interval, 1.01–6.38; *p* = 0.047).

Differences in survival can be due to imbalances between the groups or other factors unrelated to the vaccine. To evaluate this possibility, we compared the Halabi predicted survival (HPS) for the groups. The Halabi model uses seven independent prognostic markers to predict survival of men with metastatic castration-resistant prostate cancer treated with chemotherapy [21]. By comparing Halabi predicted survival between groups, one can assess potential disparities in prognostic criteria between groups of men. No difference was observed in the Halabi predicted survival for A/AB and B/O patients receiving PROSTVAC-VF (see Figure 3B), indicating no major differences in the extent of disease or aggressiveness of disease between the groups. In addition to Halabi predicted survival, we also evaluated potential correlations in the control patients. Using the Kaplan-Meier survival analysis, the A/AB and B/O control patients showed no significant difference in overall survival (see Figure 3C; *p* = 0.41) or Halabi predicted survival (see Figure 3D; *p* = 0.98). This result is consistent with prior studies demonstrating no significant differences in survival for unvaccinated prostate cancer patients based on ABO blood type [22]. Taken together, these data indicate that the survival advantage for B/O patients is unique to PROSTVAC-VF treatment.

Overall survival was also evaluated relative to the Halabi predicted survival as an indicator of the potential benefit to patients. For each individual, the survival relative to prediction was calculated by subtracting the Halabi predicted survival from the actual overall survival (OS). B/O patients had a median increase in survival of 7.5 months whereas A/AB patients had a median difference of −1.4 months (*p* = 0.03). In contrast, the median survival relative to the Halabi-model prediction in control subjects did not vary based on blood type (median difference for A/AB = −2.4 months; B/O = −0.4 months; *p* = 0.42). We also calculated the odds ratio that a PROSTVAC-VF patient would live longer than predicted (OS > HPS). Type B/O patients were about 2.8 times more likely than A/AB patients to live longer than predicted (OR = 2.75; 95% confidence interval, 1.08–6.99; *p* = 0.033). Taken together, the data suggest that the primary survival benefit relative to prediction observed for PROSTVAC-VF is derived from type B/O patients.

### Relationships between blood type, IgM to BG-A_tri_, and antibody responses to the Forssman antigen

We next compared relationships between blood type and our previously identified candidate biomarkers, pre-treatment IgM to BG-A_tri_ and antibody responses to the Forssman antigen. As one might have expected, pre-treatment IgM to BG-A_tri_ had a statistically significant correlation with blood type (A/AB vs B/O, *p* = 0.002); however, IgM to BG-A_tri_ and blood type did not stratify patients into identical groups. While many of the B/O patients had high IgM (median = 12.5; RFU on a log base 2 scale) and most of the A/AB patients had low IgM (median = 9.8), there was some crossover in the patient sets defined by blood type versus IgM to BG-A_tri_. In particular, using our previously determined cutoff for IgM to BG-A_tri_ (RFU on a log base 2 scale > 13.0), ∼10% of the A/AB patients had IgM signals that were above the cutoff and 61% of the B/O patients had IgM signals that were below the cutoff. This data exemplifies the point discussed in the introduction: IgM to BG-A and ABO blood type do not stratify the patients into identical groups. Therefore, one cannot assume that a correlation between IgM and BG-A will equate to a correlation between ABO blood type and survival.

Since IgM to BG-A and ABO blood type stratify patients into somewhat different groups, we next evaluated whether these two methods for stratifying patients were complementary. Patients with higher IgM to BG-A_tri_ tended to have improved survival by Kaplan-Meier analysis regardless of blood type. While higher IgM to BG-A_tri_ was associated with longer survival for all patients, B/O patients lived longer than A/AB at any given range of IgM to BG-A_tri_. For example, A/AB patients with IgM to BG-A_tri_ in the 10–12 range had a median survival of 20.7 months and a median survival relative to predicted survival (OS-HPS) of −3.37 months. B/O patients with IgM to BG-A_tri_ in the same range had a median survival of 26.8 months and a median survival relative to Halabi prediction of 7.7 months. Regardless of IgM to BG-A_tri_ range, there was little survival benefit relative to prediction for A/AB patients (median OS-HPS ranged from −3.37 to 1.6 months). In contrast, an improvement in median survival relative to prediction could be observed across a broad range of IgM to BG-A_tri_ for B/O patients (median OS-HPS ranging from 7.5 to 10.9 months).

**Figure 4:**
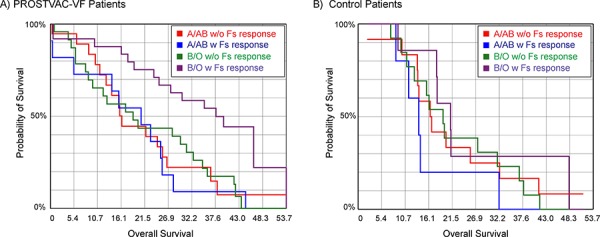
Kaplan-Meier survival curves combining blood type and antibody response to the Forssman antigen (Fs) **A.** Prostate cancer patients treated with PROSTVAC-VF (*p* = 0.003); **B.** Control patients (*p* = 0.35). Type B and O patients with antibody responses to the Forssman antigen (purple line) have improved survival in the PROSTVAC-VF arm but not the control arm.

We also examined relationships between blood type and antibody responses to the Forssman disaccharide. The Forssman antigen is a xenogenic glycolipid found on the surface of the poxviruses used in PROSTVAC-VF. We previously observed that increases in total Ig to the Forssman disaccharide of 4-fold or greater after 2–3 months on PROSTVAC-VF therapy correlate positively with survival [23]. Since the terminal disaccharide of the Forssman antigen (GalNAcα1–3GalNAcα-) and the blood group A antigen have some structural similarities (i.e. both contain a terminal GalNAc-α residue), it was possible that blood type might influence immune responses to the Forssman antigen. For example, type A/AB individuals might be less likely to have an antibody response to the Forssman antigen and/or have smaller responses than B/O individuals as a result of tolerance to BG-A-like structures. Upon analysis, however, the range of responses and median responses were nearly identical for A/AB and B/O patients (see Supporting Information, Figure S3). Thus, differences in blood type do not explain why some patients have responses to the Forssman antigen and others do not. Since Forssman responses occurred equally in A/AB and O/B patients, we next evaluated whether the correlation with survival was also independent of ABO blood type. Interestingly, Forssman responses were only associated with improved survival for B/O patients. For example, B/O patients with Forssman responses had a median survival of 37.6 months, while B/O patients without responses and A/AB patients with or without responses had median survivals of 16–20 months. These differences were readily apparent by Kaplan-Meier analysis (Figure 4A, *p* = 0.003). Similar relationships were observed for survival relative to prediction (median OS-HPS for A/AB patients without Forssman responses = −5.3 months, A/AB with responses = −1.2 months, B/O patients without responses = −0.7 months, and B/O patients with responses = 11.8 months). In the control group, blood type and Forssman response had only minimal effects on overall survival (see Figure 4B) and survival relative to prediction. Based on these results, the best survival appears to come from B/O patients who are treated with PROSTVAC-VF and produce antibody responses to the Forssman antigen after vaccination.

## DISCUSSION

ABO blood type influences numerous aspects of biology and medicine, such as susceptibility to infection by various pathogens and potential for complications due to blood transfusions. While there are a variety of methods for typing patients, standard methods are not ideal for all situations. During many clinical trials and epidemiological studies, small amounts of sera or plasma are stored for future use. These samples provide a critical resource for retrospective biomarker discovery/evaluation and mechanistic studies. In many cases, collection of additional samples is impossible, and only tiny amounts of banked sera/plasma are available to an individual researcher. Therefore, it is crucial to extract as much information as possible from the limited supplies available.

In this study, we used a glycan microarray to develop a new method to determine ABO blood types from serum antibody profiles. Although one might have expected that reverse typing using synthetic glycans would be relatively straightforward, achieving accuracies over 95% and high classification rates proved challenging. The structures and mode of presentation of the BG-A and BG-B glycans used to capture the relevant antibodies had a significant impact on the results. Simple trisaccharides provided only modest classification rates and accuracies. Tetrasaccharides conferred improved antibody capture specificity over trisaccharides, but even the best two-component tetrasaccharide systems typically provided accuracies in the 80–90% range. Ultimately, we found that the best results were obtained by using a panel of 10 BG-A and BG-B glycans, using both IgG and IgM signals, and using both high and low density glycan presentations. Using this method, accuracies over 97% and high classification rates could be consistently achieved. The accuracies for this method are comparable with standard methods, such as red blood cell agglutination assays. Importantly, the method only requires 4 μL of serum and does not require the use of red blood cells, which have limited shelf life. We envision that this assay will be useful for retrospective studies where very limited quantities of plasma/sera are available or in other cases where standard blood typing methods are not suitable.

Next, the method was used to evaluate the influence of blood type on clinical outcomes for patients treated with PROSTVAC-VF as well as control subjects. PROSTVAC-VF has demonstrated encouraging results in two phase II clinical trials and is currently in phase III trials for treatment of men with advanced prostate cancer. While promising, clinical responses vary from patient to patient, and strategies to preselect patients who are likely to respond well could have a major impact on patient care. We have previously found that IgM antibody levels to the blood group A trisaccharide correlate positively with survival. These antibodies were found to bind the BG-A antigen on the surface of the poxviral vectors that compose PROSTVAC-VF. Since type B and type O individuals would recognize the BG-A antigen as foreign and would be expected to have higher levels of antibodies to the BG-A antigen than type A and type AB patients, we hypothesized that they might also have improved overall survival. To test this hypothesis, the array method was used to assign retrospectively blood type for 109 prostate cancer patients involved in a Phase II clinical trial comparing PROSTVAC-VF versus control treatment. The blood type information was then used to evaluate relationships between blood type and survival on PROSTVAC.

Using a variety of statistical approaches, type B and O individuals demonstrated improved clinical outcomes, including longer median survival, longer median survival relative to Halabi predicted survival, and improved overall survival via Kaplan-Meier survival analysis. The largest survival benefit appears to come from B/O patients that produce an antibody response to the Forssman antigen after vaccination. The increase in survival for B/O patients relative to A/AB patients was specific to vaccination with PROSTVAC-VF. Our results suggest that screening patients for blood type could potentially provide an approach to select patients who are likely to benefit from PROSTVAC-VF treatment (e.g., type B/O patients in this study were about 2.5 times more likely than A/AB patients to live longer than 24.9 months and 2.8 times more likely than A/AB patients to live longer than expected). The implementation of this approach would be simple and inexpensive, as standard blood typing methods could be used for future work. Since blood type distributions are dependent on geographic location and race, clinical results may also vary by the location and race. For example, 70% of African-Americans in the United States have blood type B or O, whereas only 56% of Caucasians have type B or O. It is important to note that the correlation has only been assessed in a single, retrospective study; therefore, additional studies are required to validate this finding. Given the ease in which this approach could be implemented in the future (i.e. using standard blood typing methods), continued evaluation of the relationship between blood type and survival on PROSTVAC-VF is warranted.

Lastly, blood group antigens can be displayed on many different vaccines. For example, whole cell vaccines derived from human tissue can display blood group antigens, depending on the blood type of the original donor. In addition, many enveloped viruses used as vaccines are produced in cells that can biosynthesize blood group antigens. Since immune recognition of these blood group antigens would depend on the blood type of the individual receiving the vaccine, blood type may potentially influence responses and efficacy for other vaccines.

## MATERIALS AND METHODS

### Serum samples

Sera from healthy individuals of known blood type were purchased from Valley Biomedical Products and Services (Winchester, VA) (*n* = 135) and from Bioreclamation LLC (Westbury, NY) (*n* = 85). Samples were accompanied by a certification that all samples were tested in accordance with FDA regulations and found to be negative for HIV 1/2 AB, HCV AB, and non-reactive for HBSAG, HIV-1 RNA, HCV RNA, and STS. The 220 samples consisting of type O individuals (*n* = 93), type A individuals (*n* = 58), type B individuals (*n* = 32), and type AB individuals (*n* = 37). All samples were stored at −80°C or −20°C until used. For more information, see Supporting Information, Tables S1 and S2.

Analysis of relationships between blood type and survival on PROSTVAC involved patients from two previously reported, IRB-approved phase II clinical trials of PROSTVAC-VF [7, 8]. Blood type information was available in the clinical records for 8 patients with metastatic castration-resistant prostate cancer (mCRPC) who enrolled in a single-center phase II study of PROSTVAC-VF (NCT00060528) [8]. Blood type was evaluated using our assay for patients on PROSTVAC-VF (*n* = 74) and controls (*n* = 37) from a placebo-controlled, multi-center Phase II study of PROSTVAC-VF (NCT00078585) [7]. All patients received the same dose of vaccinia and fowlpox vectors, and the vaccination schedule was the same, except the subjects in the multicenter trial received an additional boost on day 14. Across all study centers, sera were obtained in serum separator tubes, processed within 4 hours, and stored at −80°C until assayed.

### Array fabrication, binding assay, and image analysis

Glycan arrays were fabricated as previously reported (for additional details, see also Supporting Information) [24, 25]. Carbohydrates were conjugated to bovine serum albumin (BSA) or human serum albumin (HSA) to produce neoglycoproteins prior to printing. Blood group determinants (A1–6, B1–6, H1–6; each with the “Oct” linker) were generously provided by Prof. Todd Lowary (University of Alberta, Canada) [26–29]. BG-A1, A2, B1, and B2 with a different linker (“Sp”) were also obtained from the Consortium for Functional Glycomics (The Scripps Research Institute, San Diego, CA). In addition to variations in structure, some glycans were printed at different densities by varying the average number of glycan molecules per molecule of BSA or HSA carrier. The number following the name abbreviation refers to the average glycan density (number of glycans/protein carrier). Averages < 8 were considered low density. The array format and assay have been validated previously with numerous antibodies and lectins [30–34]. Assay reproducibility in the context of serum antibody profiling has also been evaluated previously [25]. The microarray data discussed in this publication have been deposited in National Center for Biotechnology Information's (NCBI) Gene Expression Omnibus [GEO; [35]] and are accessible through GEO Series accession number GSE68405.

### Statistical analyses

Analyses of variance (ANOVA) were performed using Partek^®^ Genomic Suite 6.5 (St. Louis, MO) to identify BG-A and BG-B determinants that correlated best with blood type based on the calculated *p* values. Survival correlations with blood type were obtained using Kaplan-Meier survival curves and estimates, and Log-Rank *p* values are reported. Comparisons of median survival and median survival relative to Halabi predicted survival between groups were evaluated using a two-tailed *t*-test.

### Blood typing methods

Blood typing methods were developed using the normalized antibody signals to BG-A and BG-B antigens. For two component systems, the BG-A and BG-B components were systematically varied to identify optimal pairings. For each component, the signal ranges were partitioned into three groups: positive, negative, and unclassified. For example, a signal over 9.0 (RFU on a log base 2 scale) might be positive, a signal below 8.0 might be negative, and signals between 8.0 and 9.0 would be unclassified. For each BG-A or BG-B antigen, the thresholds were systematically varied. If the antibody signals to both BG-A and BG-B antigens were positive, the blood type was assigned as O. If the antibody signals to BG-A antigen were negative and to BG-B antigen positive, the blood type was assigned as A. If the antibody signals to BG-A antigen were positive and to BG-B antigen negative, the blood type was assigned as B. If the antibody signals to both BG-A and BG-B antigens were negative, the blood type was assigned as AB. If either signal was in the unclassified range, the blood type was assigned as “unclassified”. For each combination of BG-A antigen, BG-A threshold, BG-B antigen, and BG-B threshold, the classification rate and blood typing accuracy were determined. Similarly, we assigned blood types using three or more components. For multi-component systems, all components had to be in agreement.

## SUPPLEMENTAl DATA TABLES AND FIGURES


