# Supplementary material for: ABO blood type correlates with survival on prostate cancer vaccine therapy
Source: Oncotarget. 2015 Aug 18;6(31):32244–56. doi: 10.18632/oncotarget.4993 (PMC4741674; doi:10.18632/oncotarget.4993)
Supplement: Supplementary file 1 [file oncotarget-06-32244-s001.pdf]

SUPPLEMENTARY DATA

Reverse typing by agglutination

Pooled red blood cells for reverse grouping Biotestcell® A_1 and Biotestcell® B were purchased from Bio-Rad (Hercules, CA). Reverse typing was carried out using glass tubes 10 × 75mm according to the test procedure accompanied with the pooled RBCs (Biotestcell® A_1 and B) from Bio-Rad. Briefly, two drops of serum were added into each properly labeled tube (A_1 and B). One drop of Biotestcell® A_1 and B were added to each of the corresponding labeled tubes and mixed. The tubes were centrifuged for about 30 seconds at 900 × g to form a button of RBCs. The button was then gently dislodged off the tube wall to detect agglutination. Positive agglutination results indicated the presence of the corresponding antibodies, and negative agglutination results indicated the absence of the corresponding antibodies. Detailed results can be found in Table S2.

Array fabrication and binding assay

Glycan arrays were fabricated as previously reported (for additional details, see Supporting Information.[3,4] Briefly, the glycan arrays were printed on epoxide-coated glass slides in duplicate (in 28 × 28 grid for the fall array, or 14 × 14 grid for the focused array) with spot diameters of approximately 80–90 μm. The printed arrays contained various ABH and Lewis antigens (e.g. all blood group antigens type 1 through type 6), tumor-associated carbohydrate antigens, glycoproteins, *N*-linked glycans, non-human glycans, and some controls (see Supporting Data Excel file for a full list). Blood group determinants (A1–6, B1–6, H1–6; each with the “Oct” linker) were generously provided by Prof. Todd Lowary at the University of Alberta, Canada.[5–8] BG-A1, A2, B1, and B2 with a different linker (“Sp”) were also obtained from the Consortium for Functional Glycomics (The Scripps Research Institute, San Diego, CA). Carbohydrates were conjugated to BSA or HSA to produce neoglycoproteins prior to printing. In addition to variations in structure, some glycans were printed at different densities by varying the average number of glycan molecules per molecule of bovine serum albumin (BSA) or human serum albumin (HAS) carrier. The number following the name abbreviation refers to the average glycan density (number of glycans/protein carrier). Averages < 8 are considered low density and averages > 8 are considered high density. Sixteen complete arrays were printed on each slide, and the slides were stored at –20°C until used. The array format and assay have been validated previously with numerous antibodies and lectins.[9–12] Assay reproducibility in the context of serum antibody profiling had also been evaluated previously.[4]

Prior to each experiment, slides were pre-scanned (the print solution contains a soluble dye) and then images were analyzed for technical faults (e.g. missing spots). Next, a 16-well holder (Grace Bio Labs, Bend, OR) was affixed to the slide to create 16 independent array wells. The slides were blocked overnight at 4°C with 3% BSA (w/v) in PBS and washed 6 times with PBST (PBS with 0.05% (v/v) Tween 20). The reference sample and serum samples were diluted 1:50 in 3% BSA and 1% HSA in PBST, and then 100 μL of each of the diluted samples was added into two different wells in different slides (i.e. each sample was run in duplicate) and allowed to incubate at 37°C with gentle agitating (100 RPM) for 4 h. After washing 3 times with PBST, bound antibodies were detected by incubating with DyLight 549 goat anti-human IgG (3.0 μg/mL) and DyLight 649 goat anti-human IgM (3.0 μg/mL) (Jackson ImmunoResearch, West Grove, PA) in 3% HSA and 1% BSA in PBS for 2 h at 37°C with gentle agitating. After washing 7 times with PBST, the slides were removed from holders, immersed in wash buffer for 5 min, and centrifuged at 1000 RPM for 5 min. Serum samples were assayed in random order with respect to blood type and the listed blood types were blinded during data collection and processing.

Image analysis and data processing

Slides were scanned at 10 μm resolution with a Genepix 4000A microarray scanner (Molecular Devices Corporation, Sunnyvale, CA) and analyzed with Genepix Pro 6.0 software. The spots were defined as circular features with a diameter of 80 μm. The background – corrected median was used for data analysis, and any flagged spots were treated as missing. To minimize the impact of noise on our comparisons, spots with intensity lower than 150 were considered too low to accurately measure and were set to 150. The average of duplicate spots was calculated and normalized to the reference samples. A log-transformation (base 2) was applied for each slide, and the final data value was obtained from the normalized average of the data in both slides. Full IgG and IgM data can be found in the Supporting Data (Excel file)

REFERENCES

1. Kantoff PW, Schuetz TJ, Blumenstein BA, et al. Overall Survival Analysis of a Phase II Randomized Controlled Trial of a Poxviral-Based PSA-Targeted Immunotherapy in Metastatic Castration-Resistant Prostate Cancer. J Clin Oncol. 2010; 28:1099–1105.

2. Gulley JL, Arlen PM, Madan RA, et al. Immunologic and prognostic factors associated with overall survival employing a poxviral-based PSA vaccine in metastatic castrate-resistant prostate cancer. Cancer Immunol Immunother. 2010; 59:663–674.
3. Campbell CT, Zhang Y, Gildersleeve JC. Construction and Use of Glycan Microarrays. Curr Protocols Chem Biol. 2010; 2:37–53.
4. Oyelaran O, McShane LM, Dodd L, Gildersleeve JC. Profiling human serum antibodies with a carbohydrate antigen microarray. J Proteome Res. 2009; 8:4301–4310.
5. Meloncelli PJ, Lowary TL. Synthesis of ABO histo-blood group type v and VI antigens. Australian J Chem. 2009; 62:558–574.
6. Meloncelli PJ, Lowary TL. Synthesis of ABO histo-blood group type i and II antigens. Carbohydr Res. 2010; 345:2305–2322.
7. Meloncelli PJ, West LJ, Lowary TL. Synthesis and NMR studies on the ABO histo-blood group antigens: Synthesis of type III and IV structures and NMR characterization of type I-VI antigens. Carbohydr Res. 2011; 346:1406–1426.
8. Zhang D, Huang P, Zou L, Lowary TL, Tan M, Jiang X. Tulane Virus Recognizes the A Type 3 and B Histo-Blood Group Antigens. J Virol. 2015; 89:1419–1427.
9. Wang L, Cummings R, Smith D, et al. Cross-Platform Comparison of Glycan Microarray Formats. Glycobiology. 2014; 24:507–517.
10. Li Q, Anver MR, Li Z, Butcher DO, Gildersleeve JC. GalNAcα1–3Gal, a New Prognostic Marker for Cervical Cancer. Int J Cancer. 2010; 126:459–468.
11. Manimala JC, Roach TA, Li Z, Gildersleeve JC. High-throughput carbohydrate microarray profiling of 27 antibodies demonstrates widespread specificity problems. Glycobiology. 2007; 17:17C–23C.
12. Manimala JC, Roach TA, Li ZT, Gildersleeve JC. High-throughput carbohydrate microarray analysis of 24 lectins. Angew Chem. (Int Ed.)2006; 45:3607–3610.

Supplementary Table S1: Summary of blood group distributions used to develop methodology

Group	Type A	Type AB	Type B	Type O
Training Set (*n* = 60)	20 (33.3%)	10 (16.7%)	7 (11.7%)	23 (38.3%)
Test Set (*n* = 40)	12 (30%)	8 (20%)	8 (20%)	12 (30%)
Validation Set (*n* = 120)	26 (21.7%)	17 (14.2%)	18 (15%)	59 (49.1%)

Supplementary Table S2: Reverse typing by tube test of suspected samples and controls. Samples where the array data and listed blood type were in disagreement are highlighted in red

Sample #	Sample ID	AssignedBlood type	Tube TestA1 Cells	Tube TestB Cells	ReverseTyping	ArrayTyping
1	F50866–03	B	+++	-	B	B
2	F50869–08	B	++++	++++	O	O
3	F50859–09	A	-	+++	A	A
4	F50876–03	A	-	+++	A	A
5	F50862–03	O	++++	+++	O	O
6	F50867–09	O	+++	++	O	O
7	F50875–05	O	-	++	A	A
8	F50859–08	O	+++	+++	O	O
9	F50870–01	AB	-	-	AB	AB
10	F50863–01	A	-	-	AB	AB
11	F50875–04	A	-	++	A	A
12	F50875–02	O	+++	+++	O	O
13	F50872–09	A	-	+++	A	A
14	F50875–07	A	-	-	AB	AB
15	F50868–07	AB	-	-	AB	AB
16	F50860–02	A	-	+++	A	A
17	F50865–01	B	++++	-	B	B
18	F50870–07	A	-	+++	A	A
19	F50876–07	A	-	++	A	A
20	F50862–05	A	-	++++	A	A
21	F50865–07	A	-	+++	A	A
22	BRH574257	O	++++	++++	O	O
23	BRH574269	A	-	++++	A	A
24	BRH574302	B	+++	-	B	B
25	BRH574315	AB	-	-	AB	AB

Supplementary Table S3: Blood typing results for the test set (*n* = 40)

Test Set (*n* = 40)<br>BG-A Antigens	BG-B Antigens	Number of Classified	Number of Unclassified	Accuracy(Test set)
**Two components**				
BG-A3-Oct-14 (IgG)	BG-B3-Oct-17 (IgG)	35 (88%)	5 (12%)	32/35 (91%)
BG-A2-Sp -17 (IgG)	BG-B2-Sp-20 (IgG)	39 (98%)	1 (2%)	35/39 (90%)
BG-A2-Sp -17 (IgM)	BG-B2-Sp-05 (IgM)	39 (98%)	1 (2%)	33/39 (85%)
**Four components**				
BG-A3-Oct-14 (IgG)<br>BG-A2-Sp-17 (IgM)	BG-B3-Oct-17 (IgG)<br>BG-B2-Sp-05 (IgM)	32 (80%)	8 (20%)	32/32 (100%)
BG-A2-Sp-17 (IgG)<br>BG-A2-Sp-17 (IgM)	BG-B2-Sp-20 (IgG)<br>BG-B2-Sp-05 (IgM)	33 (83%)	7 (17%)	33/33 (100%)
**10 Components**				
Flow Chart (Figure 2)	Flow Chart (Figure 2)	39 (98%)	1 (2%)	39/39 (100%)

Supplementary Table S4: Summary of blood type proportions for PROSTVAC-VF study

Group	Type A	Type AB	Type B	Type O
US population	42%	4%	10%	44%
PROSTVAC-VF patients + controls (*n* = 117)	32%	8.4%	12%	47%
PROSTVAC-VF patients (*n* = 80)	30%	8.8%	10%	51%
Control patients (*n* = 37)	38%	8%	16%	38%

Supplementary Table S5: ABO Blood Types for the PROSTVAC-VF study

ID	Trial	Arm	Assigned Blood Type	Blood type from clinical records
	NCI	PROSTVAC		A
	NCI	PROSTVAC		O
	NCI	PROSTVAC		A
	NCI	PROSTVAC		O
	NCI	PROSTVAC		A
	NCI	PROSTVAC		A
	NCI	PROSTVAC		O
	NCI	PROSTVAC		AB
**1**	Multicenter	PROSTVAC	O	
**13**	Multicenter	PROSTVAC	A	
**41**	Multicenter	PROSTVAC	A	
**46**	Multicenter	PROSTVAC	O	
**74**	Multicenter	PROSTVAC	O	
**82**	Multicenter	PROSTVAC	A	
**101**	Multicenter	PROSTVAC	O	
**147**	Multicenter	PROSTVAC	A	
**152**	Multicenter	PROSTVAC	B	
**157**	Multicenter	PROSTVAC	A	
**177**	Multicenter	PROSTVAC	A	
**184**	Multicenter	PROSTVAC	A	
**192**	Multicenter	PROSTVAC	A	
**197**	Multicenter	PROSTVAC	B	
**207**	Multicenter	PROSTVAC	O	
**210**	Multicenter	PROSTVAC	B	
**218**	Multicenter	PROSTVAC	O	
**233**	Multicenter	PROSTVAC	O	
**244**	Multicenter	PROSTVAC	O	
**247**	Multicenter	PROSTVAC	B	
**252**	Multicenter	PROSTVAC	A	
**271**	Multicenter	PROSTVAC	O	
**279**	Multicenter	PROSTVAC	O	
**326**	Multicenter	PROSTVAC	O	
**357**	Multicenter	PROSTVAC	A	
**365**	Multicenter	PROSTVAC	O	
**368**	Multicenter	PROSTVAC	O	

(*Continued*)

ID	Trial	Arm	Assigned Blood Type	Blood type from clinical records
375	Multicenter	PROSTVAC	O	
380	Multicenter	PROSTVAC	AB	
407	Multicenter	PROSTVAC	O	
412	Multicenter	PROSTVAC	O	
420	Multicenter	PROSTVAC	B	
424	Multicenter	PROSTVAC	B	
432	Multicenter	PROSTVAC	O	
439	Multicenter	PROSTVAC	O	
459	Multicenter	PROSTVAC	AB	
467	Multicenter	PROSTVAC	O	
474	Multicenter	PROSTVAC	A	
482	Multicenter	PROSTVAC	O	
510	Multicenter	PROSTVAC	B	
518	Multicenter	PROSTVAC	A	
546	Multicenter	PROSTVAC	A	
563	Multicenter	PROSTVAC	O	
571	Multicenter	PROSTVAC	O	
577	Multicenter	PROSTVAC	AB	
584	Multicenter	PROSTVAC	O	
600	Multicenter	PROSTVAC	O	
608	Multicenter	PROSTVAC	O	
628	Multicenter	PROSTVAC	O	
636	Multicenter	PROSTVAC	AB	
648	Multicenter	PROSTVAC	A	
664	Multicenter	PROSTVAC	O	
670	Multicenter	PROSTVAC	O	
678	Multicenter	PROSTVAC	O	
696	Multicenter	PROSTVAC	O	
704	Multicenter	PROSTVAC	O	
738	Multicenter	PROSTVAC	O	
745	Multicenter	PROSTVAC	O	
752	Multicenter	PROSTVAC	A	
769	Multicenter	PROSTVAC	B	
776	Multicenter	PROSTVAC	A	
781	Multicenter	PROSTVAC	A	
787	Multicenter	PROSTVAC	O	
793	Multicenter	PROSTVAC	O	

(*Continued*)

ID	Trial	Arm	Assigned Blood Type	Blood type from clinical records
**802**	Multicenter	PROSTVAC	O	
**810**	Multicenter	PROSTVAC	O	
**816**	Multicenter	PROSTVAC	AB	
**822**	Multicenter	PROSTVAC	A	
**826**	Multicenter	PROSTVAC	A	
**837**	Multicenter	PROSTVAC	AB	
**842**	Multicenter	PROSTVAC	O	
**847**	Multicenter	PROSTVAC	A	
**33**	Multicenter	PROSTVAC	Unclassified	
**109**	Multicenter	PROSTVAC	Unclassified	
**9**	Multicenter	Control	O	
**21**	Multicenter	Control	O	
**29**	Multicenter	Control	O	
**65**	Multicenter	Control	A	
**89**	Multicenter	Control	O	
**117**	Multicenter	Control	O	
**127**	Multicenter	Control	O	
**136**	Multicenter	Control	O	
**162**	Multicenter	Control	O	
**170**	Multicenter	Control	B	
**221**	Multicenter	Control	AB	
**257**	Multicenter	Control	A	
**276**	Multicenter	Control	A	
**287**	Multicenter	Control	AB	
**298**	Multicenter	Control	B	
**312**	Multicenter	Control	AB	
**334**	Multicenter	Control	A	
**342**	Multicenter	Control	O	
**372**	Multicenter	Control	O	
**386**	Multicenter	Control	A	
**390**	Multicenter	Control	O	
**398**	Multicenter	Control	A	
**489**	Multicenter	Control	B	
**498**	Multicenter	Control	B	
**525**	Multicenter	Control	A	
**537**	Multicenter	Control	A	
**554**	Multicenter	Control	O	

(*Continued*)

ID	Trial	Arm	Assigned Blood Type	Blood type from clinical records
**592**	Multicenter	Control	A	
**620**	Multicenter	Control	A	
**640**	Multicenter	Control	A	
**656**	Multicenter	Control	A	
**709**	Multicenter	Control	A	
**729**	Multicenter	Control	A	
**762**	Multicenter	Control	O	
**798**	Multicenter	Control	B	
**830**	Multicenter	Control	B	
**852**	Multicenter	Control	O	

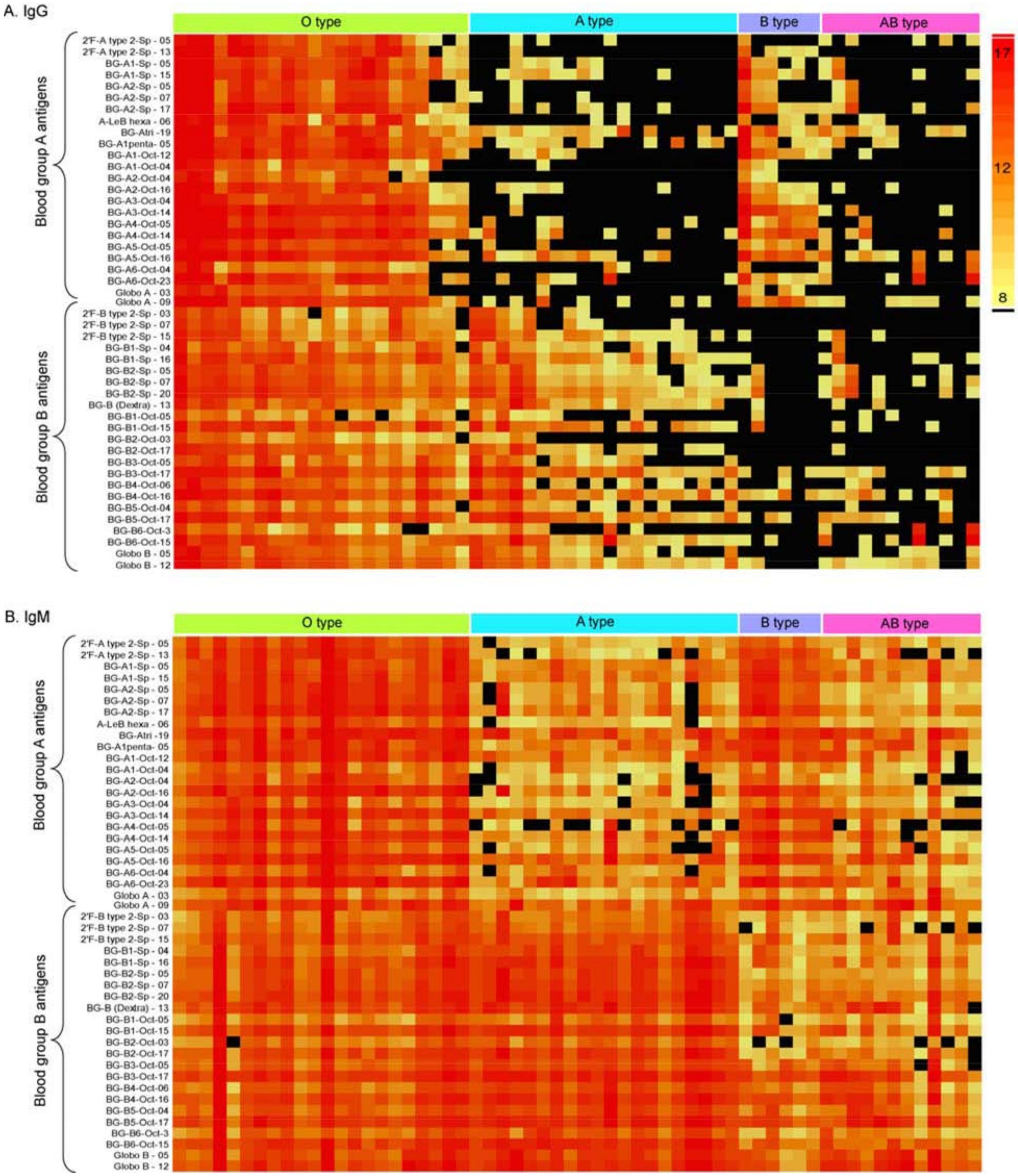

Supplementary Figure S1: Heat map representation of IgG A. and IgM B. signals to various blood group antigens. Serum samples from healthy subjects in the training set were profiled on the glycan array. Binding of IgG and IgM were detected using anti-human IgG and anti-human IgM secondary reagents. Black boxes represent no signal above background. The four samples that are potentially mis-assigned are not included in this heat map (see text for details).

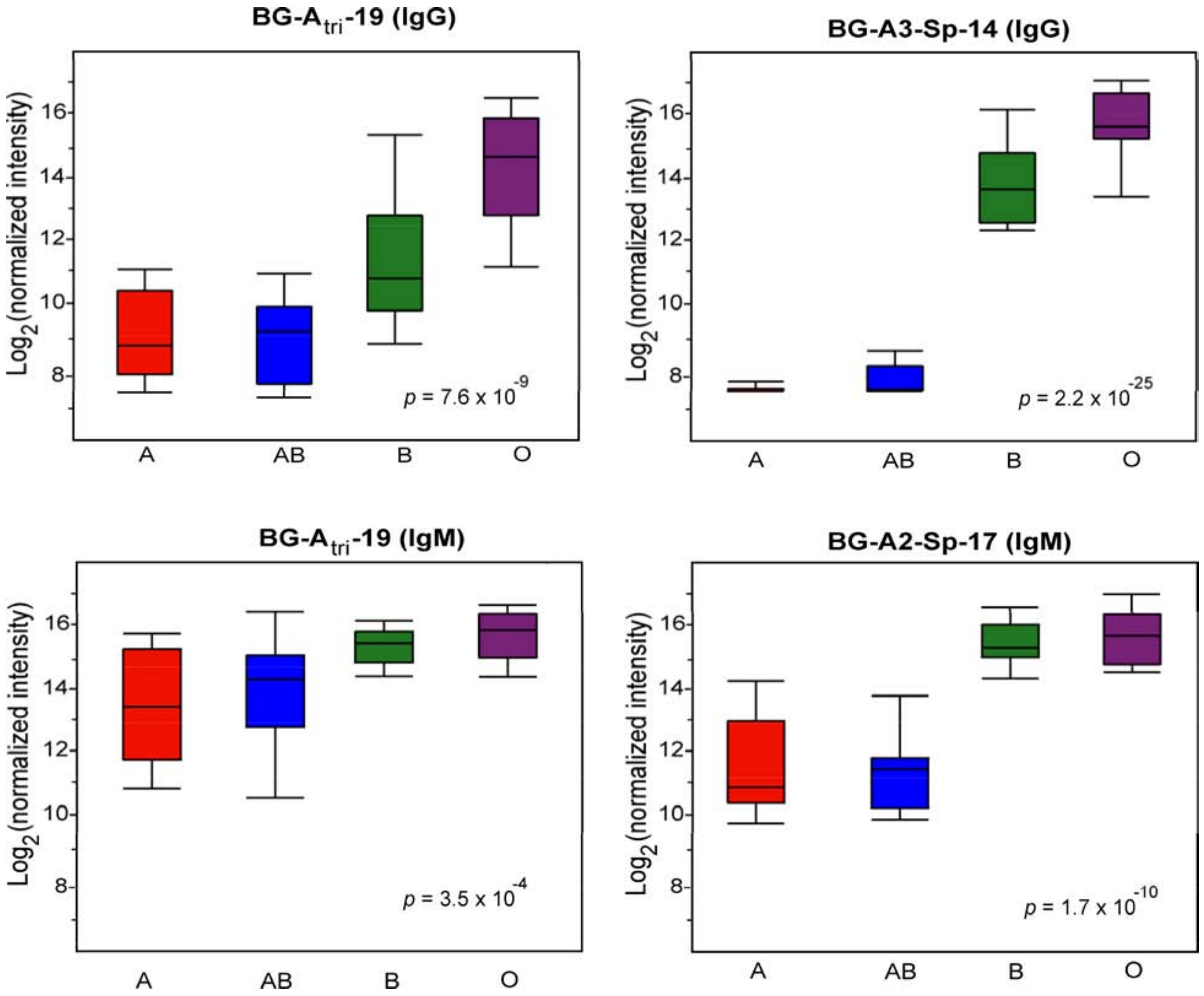

Supplementary Figure S2: Box plots showing the range of signals in healthy subjects for four blood group A variants across different blood types. The plots on the left are for the blood group A trisaccharide (BG-A_{tri}) and the plots on the right are for two blood group A tetrasaccharides. Boxes span the 25th percentile to the 75th percentile and whiskers represent the 5th and 95th percentiles.

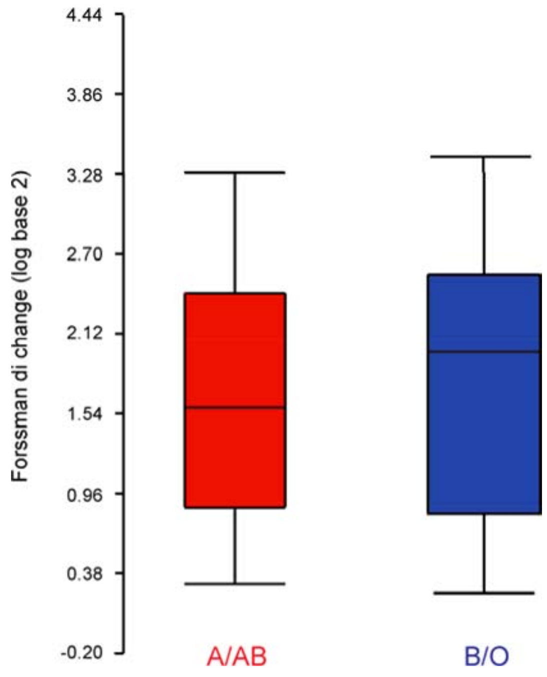

Supplementary Figure S3: Box plots comparing anti-Forssman disaccharide responses in A/AB patients (red) versus B/O patients (blue). Y-axis: changes on a log base 2 scale in total Ig to the Forssman disaccharide measured at 1:200. The box represents one standard deviation above or below the median, and the whiskers represent two standard deviations above or below the median. There is no significant difference in the distribution of Forssman responses for A/AB vs B/O patients.
